# A Simple Way of Performing Optico-Ciliary Neurotomy

**Published:** 1882-02-20

**Authors:** Jos. A. White

**Affiliations:** Virginia; Surgeon in charge of the Richmond (Va.) Eye, Ear and Throat Infirmary


### A SIMPLE WAY OF PERFORMING OPTICO-CILIARY NEUROTOMY—THE PROPOSED SUBSTITUTE FOR ENEUCLEATION.

BY JOS. A. WHITE, M. D.

Surgeon in charge of the Richmond (Va.) Eye, Ear and Throat Infirmary. Read before the Medical Society of Virginia, October 11th, 1881.

The section of the optic and ciliary nerves behind the eye-ball for the prevention of sympathetic ophthalmia after injury, was first proposed by Von Graefe; but the operation was first performed by E. Meyer and A. Weber, in 1866. Since then and more recently—in the last four or five years—it has been frequently done.

The first mode of getting at the nerves was by cutting the external rectus muscle from the sclerotic and passing the scissors through the opening thus formed. A later method was that of cutting the internal rectus. Another plan was, without cutting any of the recti, to make a meridianah incision between the internal and superior recti.

I have tried all three of these methods, and all have their objections. The cutting and re-adjusting a muscle is very troublesome. I found the opening between the upper and inner recti difficult to work through on account of the nose being in my way, and also it was objectionable because I nearly always cut an oblique muscle.

I therefore tried an opening between the upper and outer recti, as I would thus escape the oblique muscles, and have found it to work admirably in three cases so far operated on. The operation is performed as follows: A meridianal incision is made through the conjunctival and sub-conjunctival tissues from the upper border of the external rectus to the outer border of the superior rectus, thus exposing the selerotic. A strabismus hook is then inserted under each of these muscles, and with them an assistant pulls the eye down and towards the nose. A small lid elevator is then hooked under the upper lip of the incision and drawn up, thus making a large opening through which the curved scissors can be passed behind the eye-ball, and the optic and ciliary nerves cut. Knapp's double hook is then inserted into the posterior part of the sclerotic and, without any trouble, the cut end of the optic nerve and its surroundings are exposed to view at the incision. The selerotic is then carefully cleaned with the scissors—thus cutting away sections of the optic ciliary nerves. As long as any blood oozes from the opening, it is kept open. When this ceases, a conjunctival stitch is put in, and cold water dressing applied.

The three cases I have thus operated on had no pain nor protrusion of the eye following the operation, and the cornea has remained perfectly anæsthetic to this time in all of the patients, although more than six months have elapsed.

I have no where seen a suggestion to so perform this operation—the modifications having grown solely out of my difficulties in following prescribed methods. It is easier of performance than any other. The posterior part of the eye is more easily attacked from this point, and we also avoid cutting an oblique muscle; and the use of the hooks and retractor allows a free play of the scissors and a good view of the back of the eye-ball when rotated into the opening.—[Virginia Med. Monthly.

